# Qualitative and quantitative meta-analysis of acupuncture effects on the motor function of Parkinson's disease patients

**DOI:** 10.3389/fnins.2023.1125626

**Published:** 2023-05-09

**Authors:** Suying Lei, Jingqi Fan, Xin Liu, Xiaoyan Xv, Jiayan Zhang, Zipu Zhou, Lixing Zhuang

**Affiliations:** ^1^The First Clinical School of Medicine, Guangzhou University of Chinese Medicine, Guangzhou, Guangdong, China; ^2^Guangzhou University of Chinese Medicine, Guangzhou, Guangdong, China; ^3^The First Affiliated Hospital of Guangzhou University of Chinese Medicine, Guangzhou, Guangdong, China

**Keywords:** acupuncture, Parkinson's disease, dose-response, meta-analysis, motor function

## Abstract

**Objective:**

To explore the association between acupuncture sessions and its effects on the motor function of Parkinson's Disease (PD).

**Methods:**

Eight databases and two clinical trials registries were searched from inception to August 2022. Randomized controlled trials (RCTs) that compared acupuncture with sham acupuncture, or antiparkinsonian drugs, were included. After qualitative meta-analysis, a non-linear meta regression approach with restricted cubic spline was used to investigate the dose-response relationship between acupuncture sessions and their efficacy on the Unified Parkinson's Disease Rating Scale Part III (UPDRS-III) score. Subgroup meta-analysis was performed of the included studies according to the weekly acupuncture frequency. And finally, the included studies containing the determination of intermediate efficacy were compared.

**Results:**

Of the 268 citations screened, 16 studies (462 patients of PD) were included. The qualitative meta-analysis showed that the acupuncture group had better effect on UPDRS-III scores than the control group. And the quantitative meta-analysis suggested that acupuncture dose was correlated with the reduction of UPDRS-III score in PD patients with motor symptoms. In subgroup analysis, on the one hand, when the frequency of acupuncture was no more than 3 times a week, with the increase of acupuncture session, the changes of UPDRS-III score decreased and then increased (*P* = 0.000). On the other hand, when acupuncture for more than 3 times a week and the dose of acupuncture treatment was <60 times, the changes of UPDRS-III score increased with the increase of acupuncture dose, but the score stopped to decrease if the dose continued to increase (*P* = 0.020). The comparative analysis of two quantitative RCTs found that the score improvement was more significant at the higher weekly acupuncture frequency.

**Interpretation:**

This study found that when treating PD patients with motor symptoms, acupuncture treatment may need to reach a certain dose to obtain better therapeutic effect and excessive acupuncture stimulation may cause the body to develop a certain tolerance. However, the above results still need to be verified by more high-quality clinical studies. The protocol was registered on PROSPERO International Prospective Register of Systematic Reviews (CRD42022351428).

## 1. Introduction

Parkinson's disease (PD) is a common neurodegenerative disorder primarily characterized by the deterioration of motor activities, including tremor at rest, bradykinesia, rigidity and postural instability (Pajares et al., 2020). The incidence of PD has increased rapidly in the past two decades. In 2016, the number of PD patients worldwide has reached 61 million (Dorsey et al., 2018). Although many treatments have been carried out to treat PD, none of them, alone or in combination, are capable of halting the disease progression in the long run (Sola et al., 2022).

In traditional Chinese medicine (TCM), PD symptom was first described as shaking palsy by Huangdi Neijing (ca. 100 A.D.), and TCM has played an indispensable role in medical care of PD patients for thousands of years. Many doctors and patients worldwide now use acupuncture, a technique of TCM that originated 2000 years ago (Ma et al., 2016), as a treatment for PD to alleviate its motor and non-motor symptoms. However, the international recognition of acupuncture treatment of PD is not high enough, for evidence-based medical literature related to acupuncture treatment of PD is mainly published in Chinese journals, and their quantity and quality are not high. In recent years, more and more randomized controlled trials (RCTs) have focused on acupuncture therapy for PD and many evidences have confirmed that acupuncture can alleviate motor symptoms and non-motor symptoms in patients with PD (Xia et al., 2012; de Amorim Aroxa et al., 2017; Wu et al., 2021). Since there are many different non-motor symptoms in PD and the number of RCTs related to each symptom is small, it is not advisable to directly combine related RCTs, so this study focused on motor symptoms. A randomized controlled crossover study found that a single acupuncture treatment can significantly improve the motor symptoms of PD, including gait speed, gait cadence, support base width, medio-lateral oscillation, left-right step length, and the like (Pereira et al., 2021). Several recently published qualitative meta-analyses suggested that acupuncture-related therapies combined with conventional medication showed a moderate or large effect on movement function in patients with PD (Lee and Lim, 2017; Kwon et al., 2021; Wen et al., 2021), and compared with using conventional medication alone, the combination of acupuncture in the treatment of PD, to some extent, can also improve clinical safety (Liu et al., 2017).

Exploring the relationship between different exposure levels and the development of disease is a research hotspot in epidemiology (Bauer et al., 2020). As early as more than 10 years ago, some researchers has recognized the “dose-response” in acupuncture, and they proposed the definition of dose should include the physical procedures applied in each session, using one or more needles, taking account of the patient's resulting perception (sensory, affective and cognitive) and other responses (including motor) (White et al., 2008). Research examining an adequate dose of acupuncture therapy with optimal intervention parameters and time table has also long been neglected and is now urgent (Ma, 2020). Recently, a dose-response meta-analysis on major depressive disorder have shown that acupuncture sessions were strongly correlated with its efficacy (Xu et al., 2022). On acupuncture for PD, to date, there is no systematic review of the dose-response relationship between acupuncture sessions and its efficacy on PD, but some RCTs have confirmed that the increase of acupuncture sessions may be related to the degree of remission in motor symptoms (Lei et al., 2016). In order to provide reference for the dose-response relationship and the optimal dose of acupuncture in treating motor symptoms of Parkinson's disease, we systematically collected relevant clinical RCTs literature, and made a qualitative and quantitative meta-analysis on acupuncture treatment for motor symptoms of PD patients.

## 2. Subjects/materials and methods

### 2.1. Methods

The protocol was registered on PROSPERO International Prospective Register of Systematic Reviews (CRD42022351428). This dose-response meta-analysis was reported according to the Preferred Reporting Items for Systematic Reviews and Meta-Analyses statements (Moher et al., 2009).

#### 2.1.1. Search strategy

Eight databases (Pubmed, medline (*via* Embase), Cochrane, Embase, China National Knowledge Infrastructure (CNKI), China Biomedical Literature Database (CBM), VIP Database (VIP) and WanFangData) and two clinical trial registries (ClinicalTrials.gov and Chinese Clinical Trial Registry) were searched for RCTs published from the database inception to August 2022. Various combinations of Medical Subject Headings (MeSH) and non-MeSH terms were used, including “Parkinson's disease”, “Parkinson disease”, “paralysis tremor”, “Parkinsonian”, “acupuncture”, “acupuncture”, “warm acupuncture”, “electric acupuncture”, “head acupuncture”, “body acupuncture” “Abdominal acupuncture”, “acupuncture”, “acupuncture”, “moxibustion”, “motor symptoms”, “random”, “RCT”. Language, study population, or country restrictions were not applied. The specific search strategy is provided in Table 1.

**Table 1:** Search strategy for PubMed.

**Number**	**Search terms**
#1	“Acupuncture”[Mesh] OR “Acupuncture Therapy”[Mesh]
#2	“Pharmacopuncture”[Title/Abstract] OR “Therapy, Acupuncture”[Title/Abstract] OR “Pharmacoacupuncture Therapy”[Title/Abstract] OR “Therapy, Pharmacoacupuncture”[Title/Abstract] OR “Acupuncture Treatment”[Title/Abstract] OR “Acupuncture Treatments”[Title/Abstract] OR “Treatment, Acupuncture”[Title/Abstract] OR “Pharmacoacupuncture Treatment”[Title/Abstract] OR “Treatment, Pharmacoacupuncture[Title/Abstract] OR “Acupotomy”[Title/Abstract] OR “Acupotomies”[Title/Abstract]
#3	#1 or #2
#4	“Parkinson Disease”[Mesh]
#5	“Lewy Body Parkinson's Disease”[Title/Abstract] OR “Parkinson's Disease, Idiopathic”[Title/Abstract] OR “Parkinson's Disease, Lewy Body”[Title/Abstract] OR “Parkinson Disease, Idiopathic”[Title/Abstract] OR “Parkinson's Disease”[Title/Abstract] OR “Idiopathic Parkinson Disease”[Title/Abstract] OR “Lewy Body Parkinson Disease”[Title/Abstract] OR “Primary Parkinsonism”[Title/Abstract] OR “Parkinsonism, Primary”[Title/Abstract] OR “Paralysis Agitans”[Title/Abstract]
#6	#4 or #5
#7	“motor symptom”[Title/Abstract]
#8	“randomized controlled trial”[Publication Type] OR “randomized”[Title/Abstract] OR “placebo”[Title/Abstract]
#9	#3 and #6 and #7 and #8

#### 2.1.2. Inclusion and exclusion criteria

All RCTs with eligible intervention(s) and outcome(s) for motor symptoms of PD published in Chinese or English languages were included. The included RCTs should strictly follow the principle of randomization, but because acupuncture therapy is difficult to strictly follow blinding and placebo control, there were no strict requirements. The included subjects should meet the diagnostic criteria for PD (Litvan et al., 2012; Yu et al., 2017).

Studies with more interventions, such as rehabilitation therapy or traditional Chinese medicine, were excluded. The included patients did not change their medication regimen 1 month before or during the intervention. There were no distinctions on manipulation methods, acupoint selection, needle retaining time and follow-up period. Moreover, no restrictions were set for comparators.

The primary outcome was the Unified Parkinson's Disease Rating Scale Part III (UPDRS-III), version 3.0. The UPDRS-III scores were rated before and after acupuncture. The improvement rate was defined as the percentage change in UPDRS-III score compared to the baseline to assess the effectiveness of acupuncture.

### 2.2. Studies selection process

**Figure 1:**
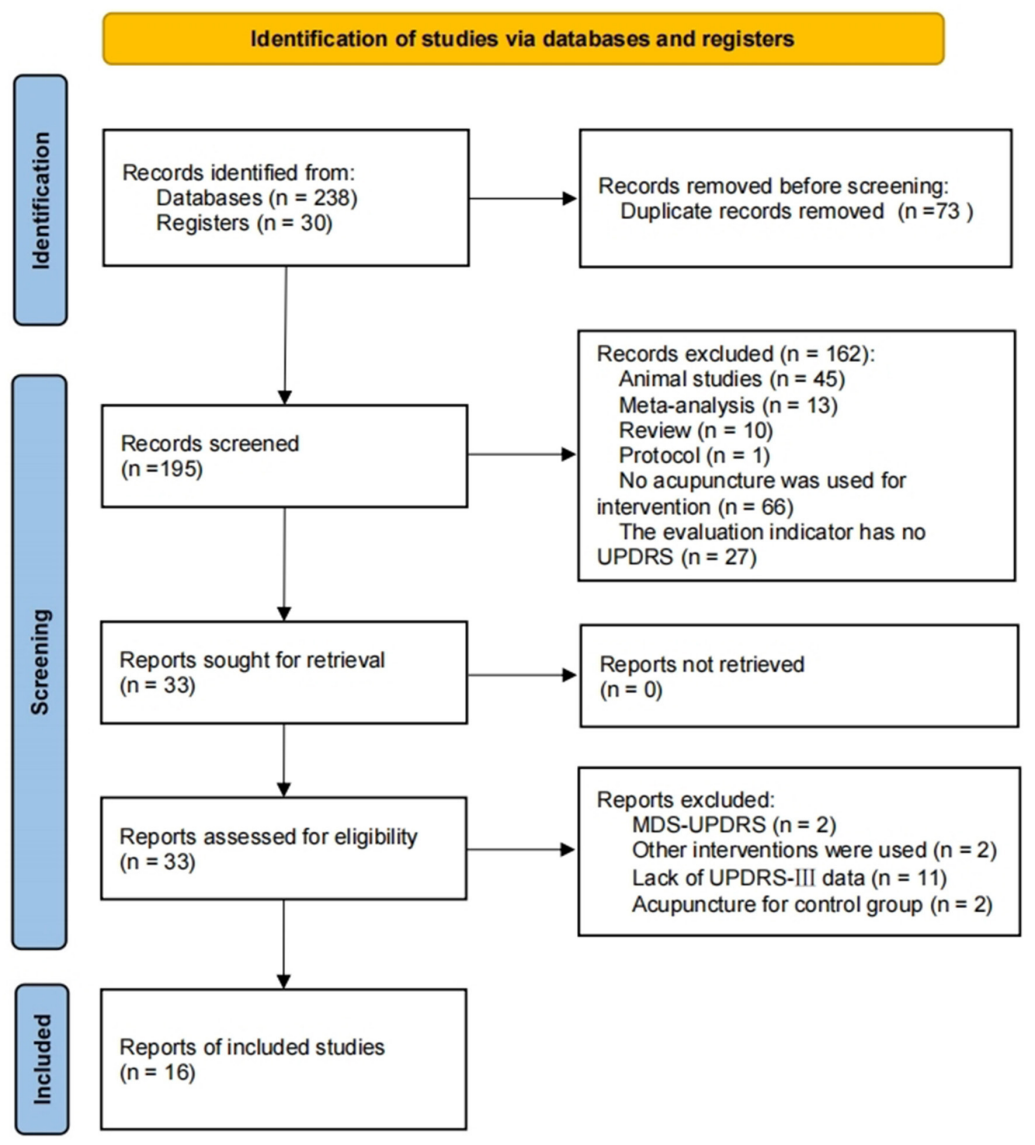
Flowchart of study selection for inclusion.

Figure 1 illustrates the process of studies selection. Firstly, the retrieved literatures were imported into NoteExpress 3.6.0.9220, and its automatic review function was used to remove duplicate articles. Secondly, two independent investigators (LSY and XXY) sifted out unrecognized duplicates, including duplications from different publications and multilingual publications, as well as reports on different aspects of the same research. These two investigators then screened the titles and abstracts of the articles to select eligible studies based on the type of research, interventions/comparators and outcomes. Thirdly, a fulltext assessment was performed by two investigators (LX and FJQ) to exclude articles according to the exclusion criteria. Any discrepancies were resolved by consensus or consulting a fifth investigator (ZLX).

### 2.3. Risk of bias assessment

The methodological quality of eligible trials was measured *via* the Cochrane scoring system (Higgins et al., 2011). Each included study was evaluated by two independent researchers (LSY and XXY) based on seven items, including the method of random sequence generation, concealment of treatment allocation; blinding (participants, healthcare providers, data collectors, outcome assessors, and data analysts); infrequent missing outcome data and free from selective outcome reporting. The evaluation was graded into one of the three categories: low risk, high risk or unclear risk of bias. Any dissent occurs in the procedures were judged by a third investigator (FJQ) after the cross-checking of the study assessment.

### 2.4. Data extraction

Data were independently extracted from eligible studies by two researchers (LSY and XXY), and a chief investigator (FJQ) made a final assessment on any inconsistencies to reach a consensus. An electronic data-extraction form was designed, including study characteristics (author information, publication year, title, study design, etc.); participant details (age, gender, duration, diagnosis, etc.); method of intervention/control (number of treatments, frequency, etc.) and outcomes (types of primary and secondary outcome measures, mean and standard deviation (SD) of UPDRS-III scores, adverse event, etc.).

### 2.5. Data synthesis and statistical analysis

Qualitative meta-analysis was performed using Review Manager 5.4 and if the therapeutic effect of acupuncture group is significantly better than that of control group, quantitative meta-analysis will be conducted. The dose-response relationship between acupuncture sessions and reduction in UPDRS-III scores were established in Stata 16.0 software (Stata Corp., College Station, TX, USA), using the robust-error meta-regression (REMR) method (Xu and Doi, 2018), Each studies was treated as a cluster, and the mean changes in UPDRS-III scores were used as effect estimations while the acupuncture session as “dose” in the meta-regression analysis. The effects of different acupuncture doses on the difference of UPDRS-III score before and after the experiment were explored. Meta-regression models were fitted to the data according to the “one-stage” framework of REMR method. The non-linear relationship was approximated using restricted cubic spline (RCS). Three knots were set to place splines inserting values for the mean changes of UPDRS-III score, to ensure that the cubic spline was restricted to be linear at the tails of the function. Modeling of potential non-linear relationships was tested by restricting the regression coefficient to zero and a *P*-value < 0.1 (Wold, 1974).

Previous studies have shown that the efficacy of acupuncture can be affected by many factors (Lin, 2016; Zhang et al., 2021). Among these factors that may influence the efficacy of acupuncture, weekly acupuncture frequency was selected for study. Moreover, a comparative analysis was performed among the studies with documented intermediate efficacy in the included studies and line plots were drawn by SigmaPlot 12.0. Theoretically, there were methodological blind spots for regression publication bias analysis, because the dose-response relationship in this study was essentially based on non-linear regression theory (Xuchang et al., 2015). Due to the lack of valid measurement methods for one-stage dose-response meta-analysis, the heterogeneity was roughly estimated by I-R^2^. Two-sided *p*-values < 0.05 were statistically significant.

## 3. Results

### 3.1. Study selection

As shown in Figure 1, the electronic search yielded 268 unique records. Among these, 73 studies were excluded due to duplication. After that, 162 studies were excluded due to the following reasons: animal studies (*n* = 45), meta-analysis (*n* = 13), review studies (*n* = 10), protocol (*n* = 1), no acupuncture was used for intervention (*n* = 66) and the evaluation indicator has no UPDRS (*n* = 27). Consequently, 33 relevant studies remained for retrieval and all the articles were retrieved. After assessing for eligibility, 17 studies were removed due to lack of required information, including 2 studies (Toosizadeh et al., 2015; Jia et al., 2022) used MDS-UPDRS (Goetz et al., 2008) in evaluating motor function in PD. Finally, 16 studies were included in the current study.

### 3.2. Characteristics of the included studies

The features of the included studies were shown in Table 2. There were 16 trials with a total of 462 participants. The studies were conducted from 2006 to 2022 and their sample size varied from 14(Li, 2016) to 50 (Li, 2015) participants, with age ranged from 49.33 (Wu, 2006) to 69.48 (Li, 2015). Three studies lacked of the mean duration of patients (Wu, 2006; Li, 2015; Nazarova et al., 2022) and one study not identified its mean age (Nazarova et al., 2022). At the baseline, the UPDRS-III score was ranged from 17.47 (Nazarova et al., 2022) to 43.40 (Jiang et al., 2006).

**Table 2:** Characteristics of included studies.

**Study ID**	**Sample size**	**Frequency (week)**	**Session**	**Sex (man)**	**Age (year)**	**Duration (year)**	**UPDRS-III_baseline**
Li, 2015	50	5	60	30	69.48 ± 5.42	2.18^*^	20.28 ± 2.93
Li, 2016	14	2	24	8	62.85 ± 5.00	5.03 ± 4.73	26.00 ± 15.07
Wu, 2006	35	4	30	17	49.33 ± 13.71	2.58 ± 0.71	24.17 ± 11.01
Yang, 2009	31	5	80	19	62.21 ± 4.48	4.98 ± 2.86	25.25 ± 11.14
Zhang, 2020	25	5	20	15	67.68 ± 5.105	2.07^*^	33.08 ± 6.21
Zhuang and Zhuang, 2012	31	5	40	19	61.21 ± 4.48	5.28 ± 3.44	25.25 ± 11.14
Chen et al., 2012	30	6	36	19	61.93 ± 3.67	6.40 ± 2.15	34.88 ± 8.49
Qiu, 2021	26	6	48	15	61.23 ± 6.51	2.72^*^	23.38 ± 10.52
Jiang et al., 2006	15	5	30	8	56.80 ± 10.93	6.9 ± 2.64	43.40 ± 11.9
Nazarova et al., 2022	15	2	16	9	66.90 ± 7.80	–	17.47 ± 7.51
Kong et al., 2018	19	2	10	6	62.90 ± 9.70	–	27.10 ± 13.7
Xu et al., 2020	33	4	32	15	61.95 ± 9.77	3.26 ± 2.32	35.61 ± 10.02
Han et al., 2022	48	5	80	30	61.00 ± 3.00	4.40 ± 2.60	36.04 ± 6.12
Wu, 2006	31	2	6	16	62.03 ± 10.23	2.88^*^	27.81 ± 7.04
Xu, 2021	29	2	6	12	69.28 ± 6.48	6.09^*^	30.59 ± 12.91
Zhang et al., 2018	30	3	24	–	–	–	27.53 ± 9.13

* Missing standard errors.

### 3.3. Risk of bias

**Figure 2:**
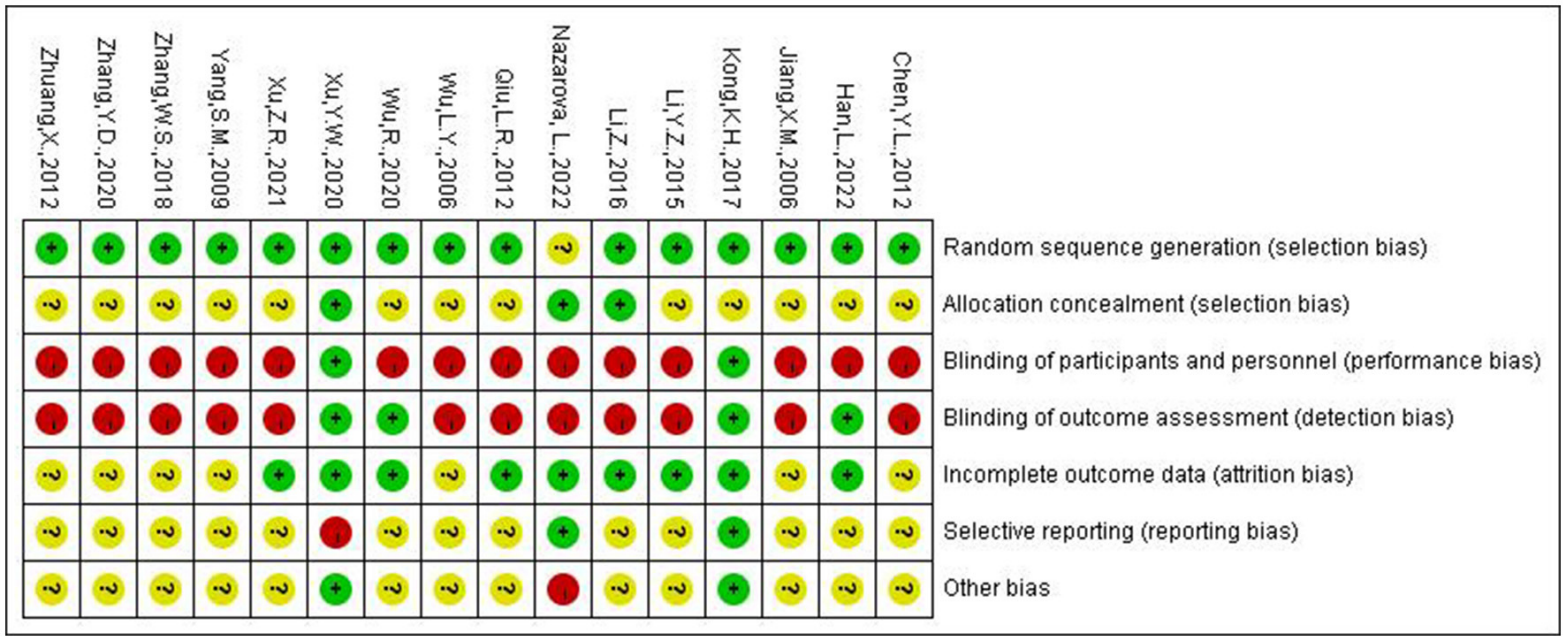
Risk of bias summary graph.

Figure 2 presented the risk of bias in each study. One major limitation was the low levels of reported blinding for participants, investigators, and outcome assessors. Among the included studies, only 1 study (Nazarova et al., 2022) did not specify the randomization methods; 3 studies (Toosizadeh et al., 2015; Li, 2016; Xu et al., 2020) mentioned assignment concealment; 4 studies (Jiang et al., 2006; Kong et al., 2018; Xu et al., 2020; Yang et al., 2020) specified assessor blinding and 2 studies (Kong et al., 2018; Xu et al., 2020) blinded patients in addition to assessor blinding; 9 studies (Jiang et al., 2006; Li, 2015, 2016; Kong et al., 2018; Xu et al., 2020; Yang et al., 2020; Qiu, 2021; Xu, 2021; Nazarova et al., 2022) reported dropout, loss to follow-up; 2 studies (Kong et al., 2018; Xu et al., 2020) has been registered, and one of the studies (Xu et al., 2020) did not report the registered primary outcome indicators such as Berg Balance Scale, PDQ-39, so that the selective reporting outcome was high risk. 2 studies (Kong et al., 2018; Xu et al., 2020) identified no conflict of interest, 1 study (Nazarova et al., 2022) lacked of baseline data and diagnostic criteria.

### 3.4. Side effects

Among the 16 included literatures, only 2 studies (Zhang, 2020; Hong et al., 2022) reported halo needle. 3 studies (Jiang et al., 2006; Chen et al., 2012; Han et al., 2022) reported that the incidence of side effects like dizziness, vomiting and insomnia in acupuncture combined with conventional medicines were lower than those in conventional medicines alone.

### 3.5. Qualitative meta analysis

**Figure 3:**
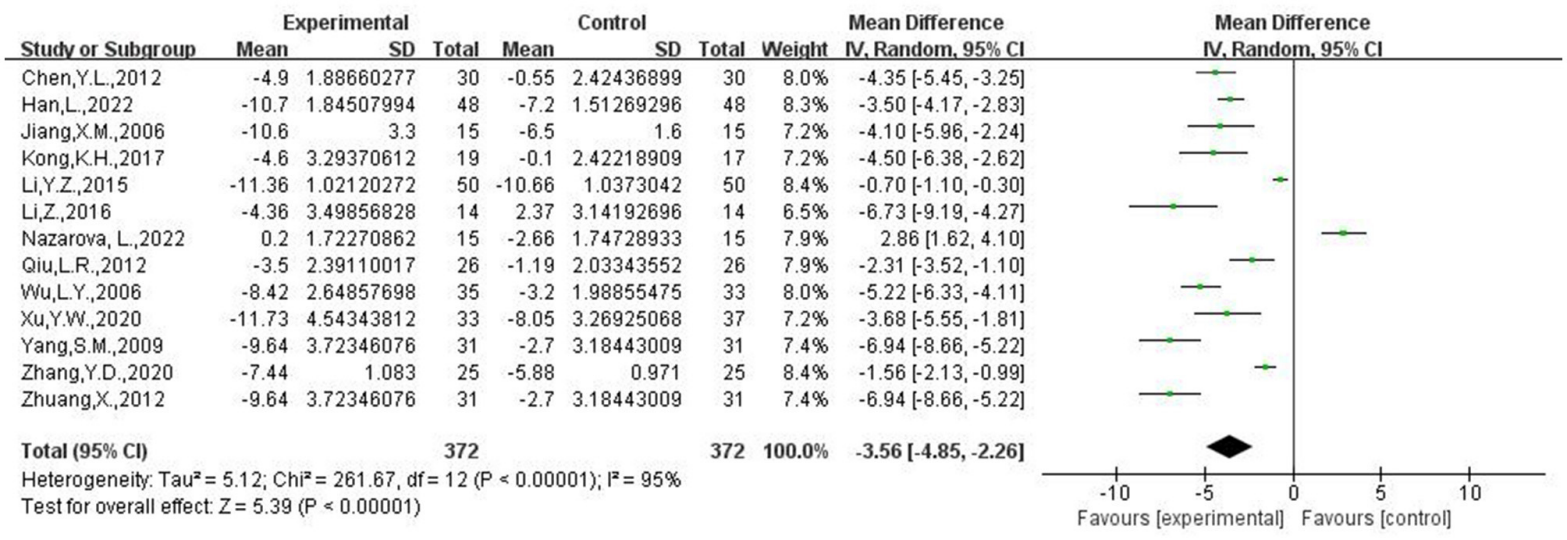
Meta-analysis of the difference between acupuncture group and control group before and after treatment.

A total of 13 studies (Jiang et al., 2006; Wu, 2006; Yang, 2009; Chen et al., 2012; Zhuang and Zhuang, 2012; Li, 2015, 2016; Kong et al., 2018; Xu et al., 2020; Zhang, 2020; Qiu, 2021; Han et al., 2022; Nazarova et al., 2022) were included after the removal of 3 studies (Zhang et al., 2018; Yang et al., 2020; Xu, 2021) in which acupuncture was used in both the experimental group and the control group. Figure 3 showed significant heterogeneity between the acupuncture group and the control group (*I*^2^ = 95%, *P* < 0.00001), so the random effects model was used, and the results showed that the acupuncture group had a better effect on the UPDRS-III score of PD patients than the control group [MD = −3.56, 95% CI (−4.85, −2.26), *P* < 0.00001].

### 3.6. Dose-response meta analysis

#### 3.6.1. Relationship between acupuncture session and the change of UPDRS-III score

**Figure 4:**
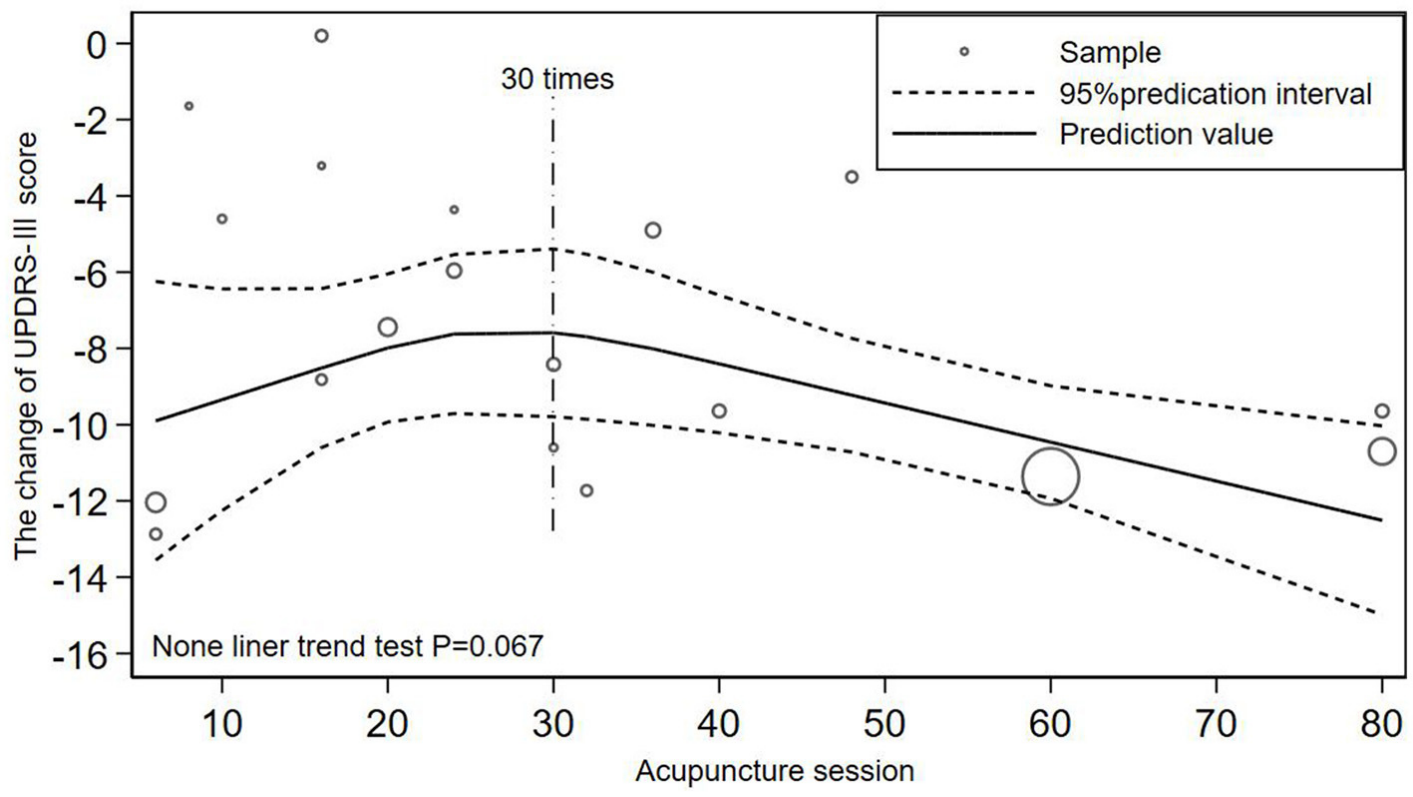
Non-linear relationship between acupuncture session and changes of UPDRS-III score.

16 literatures were included (Jiang et al., 2006; Wu, 2006; Yang, 2009; Chen et al., 2012; Zhuang and Zhuang, 2012; Li, 2015, 2016; Kong et al., 2018; Zhang et al., 2018; Xu et al., 2020; Yang et al., 2020; Zhang, 2020; Qiu, 2021; Xu, 2021; Han et al., 2022; Nazarova et al., 2022) to illustrate the dose-response relationship between the number of acupuncture sessions and the change of the UPDRS-III score. The results in Figure 4 showed that there was a non-linear dose-response relationship between acupuncture session and changes of UPDRS-III score. The change of UPDRS-III score decreased and then increased with increasing acupuncture session (*P* = 0.067; Figure 4). Dose of 30 times was the inflection point of UPDRS-III score changes.

#### 3.6.2. Subgroup analysis of the weekly acupuncture frequency

**Figure 5:**
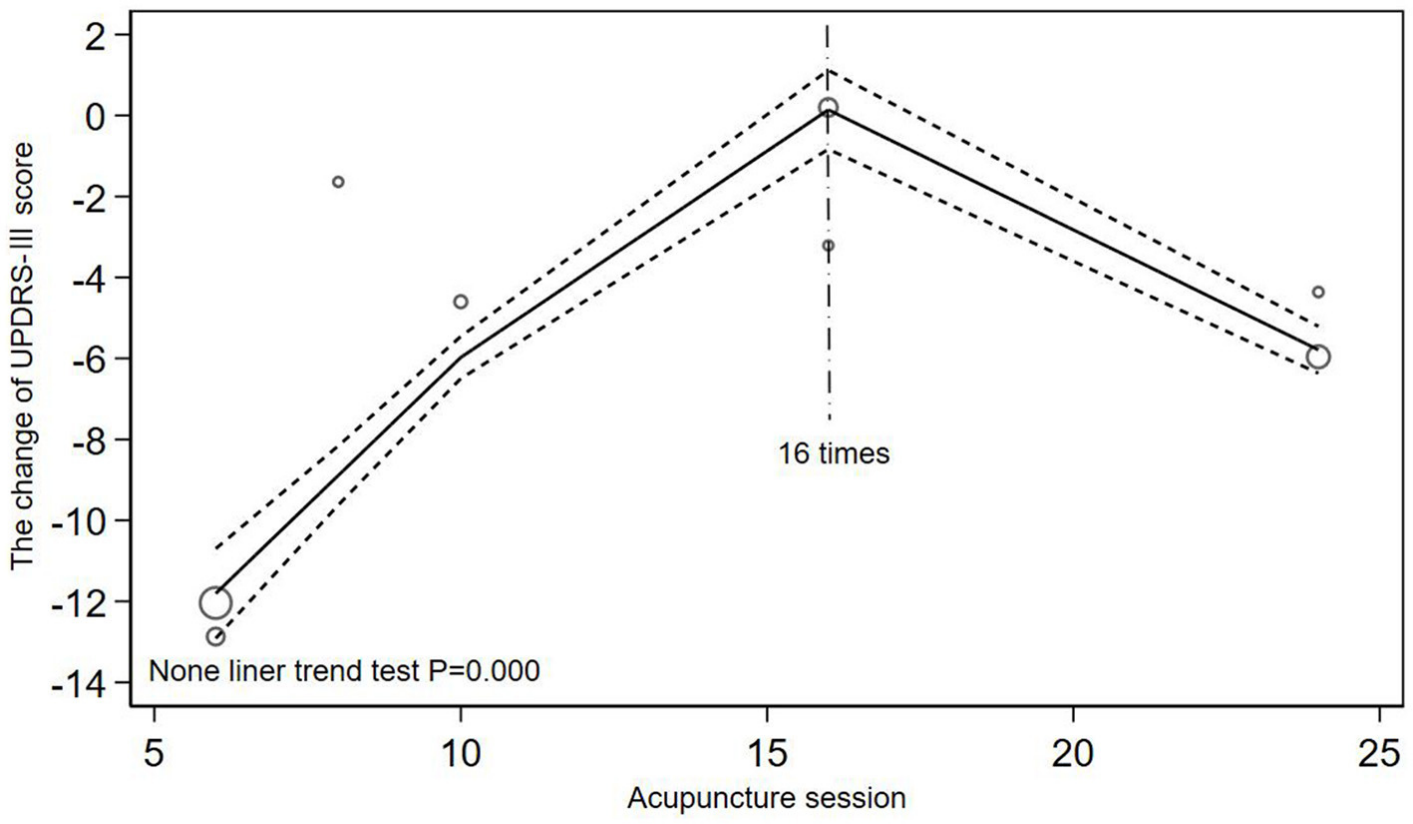
Inverted V-shaped relationship between acupuncture dose and the changes of UPDRS-III score when acupuncture for <3 times a week.

**Figure 6:**
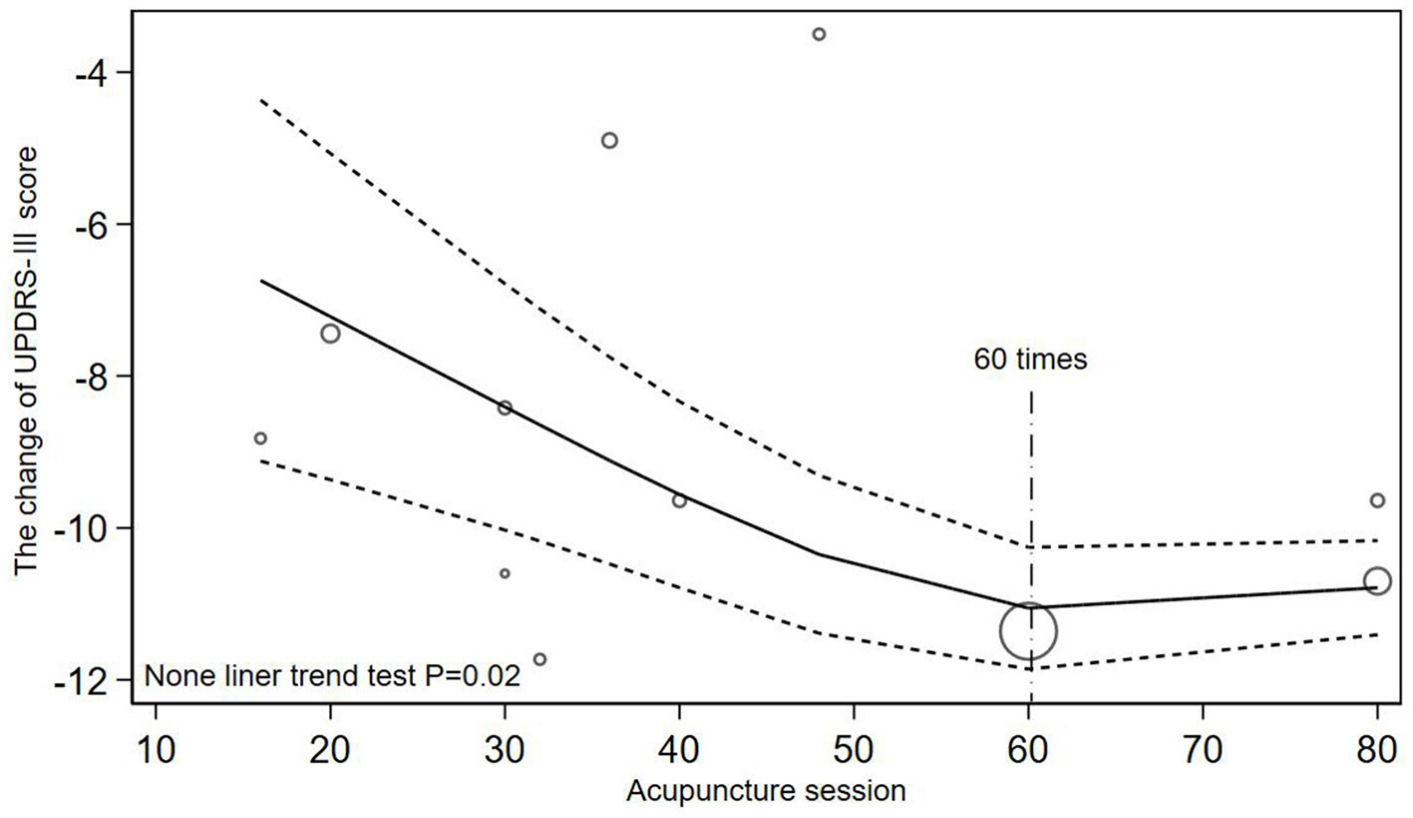
Non-linear relationship between acupuncture dose and the changes of UPDRS-III score when acupuncture for more than 3 times a week.

The included studies were categorized into two groups with acupuncture <3 times a week (Toosizadeh et al., 2015; Li, 2016; Kong et al., 2018; Zhang et al., 2018; Yang et al., 2020; Xu, 2021; Jia et al., 2022; Nazarova et al., 2022) and acupuncture more than 3 times a week (Jiang et al., 2006; Wu, 2006; Yang, 2009; Chen et al., 2012; Zhuang and Zhuang, 2012; Li, 2015; Xu et al., 2020; Zhang, 2020; Qiu, 2021; Han et al., 2022). Figures 5, 6 illustrated a non-linear dose-response relationship of the two subgroups, respectively. Figure 5 showed the subgroup with acupuncture <3 times a week. With the increase of acupuncture session, the changes of UPDRS-III score decreased and then increased and the inflection point appeared at 16 times of acupuncture session (*P* = 0.000). The non-linear dose-response relationship of subgroup with acupuncture more than 3 times a week was demonstrated in Figure 6 (*P* = 0.020). When the dose of acupuncture treatment was <60 times, the changes of UPDRS-III score increased with the increase of acupuncture session, but the score stopped to decrease if the dose continued to increase.

#### 3.6.3. Comparative analysis of two quantitative RCTs

**Figure 7:**
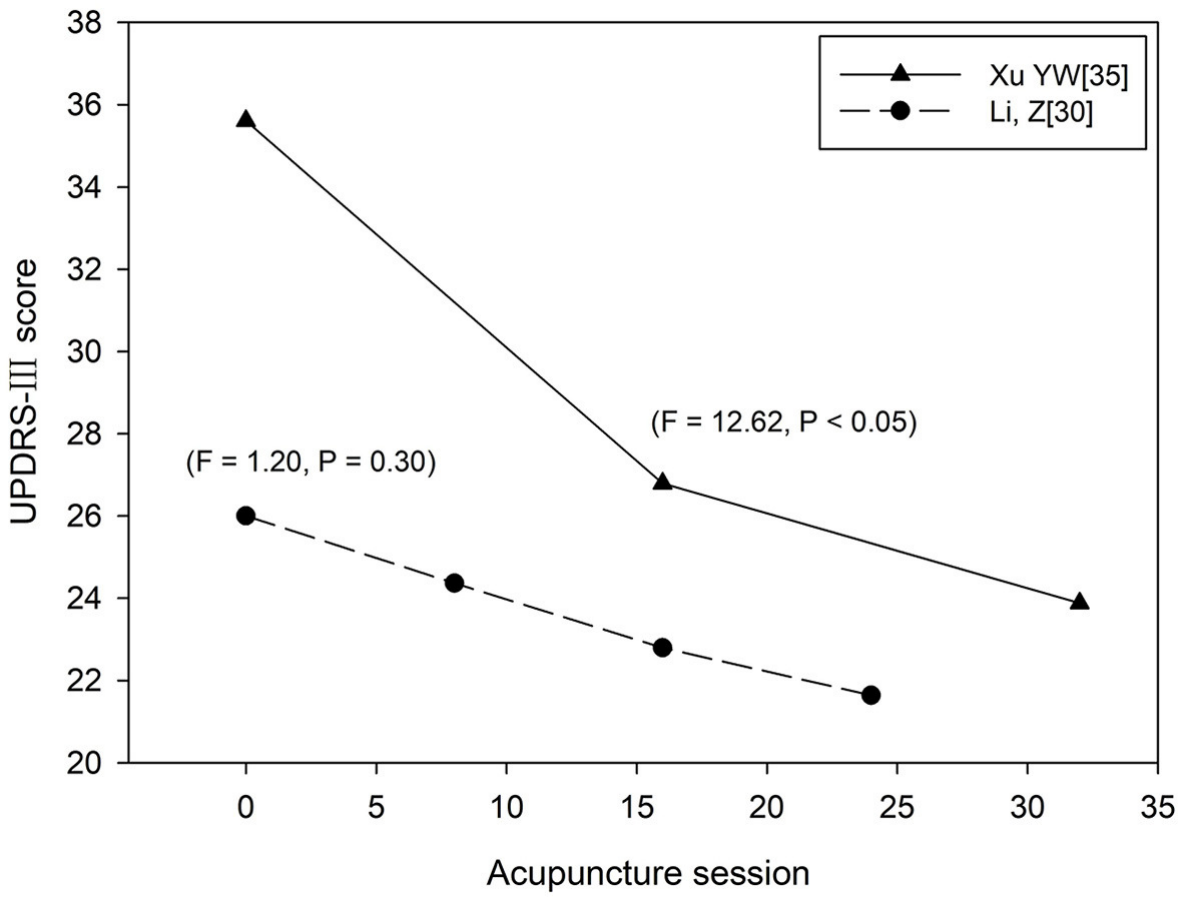
Comparative analysis of two quantitative RCTs.

Two of the included studies recorded the scores at different stages of the treatment process (Li, 2016; Xu et al., 2020). As shown in Figure 7 (Li, 2016), by treating acupuncture twice a week and comparing the UPDRS-III score after 0, 8, 16 and 24 acupuncture sessions, Li found no significant difference within the group before and after treatment (*P* = 0.30). The multicentre RCT of acupuncture for PD patients by Xu showed that UPDRS-III score varied significantly at session of 0,16, and 32 when acupuncture treatment applied by four times a week (*P* < 0.05).

## 4. Discussion

### 4.1. Main findings

Acupuncture has been used to treat PD patients since ancient times (Ma et al., 2016). Madopar or other dopaminergic drugs combined with acupuncture have been used to treat PD patients by physicians aiming to reduce side effects and increase therapeutic effectiveness (Xia et al., 2012; de Amorim Aroxa et al., 2017; Wu et al., 2021). Many systematic reviews have found that acupuncture showed a moderate or large effect with moderate or high certainty evidence in many diseases or conditions, including neurological diseases (Lee and Lim, 2017; Kwon et al., 2021; Wen et al., 2021; Lu et al., 2022). Recently, a quantitative meta-analysis on major depressive disorder found that the number of acupuncture sessions was correlated with a reduction in HAMD score in patients with MDD (Xu et al., 2022). On acupuncture for PD patients, to date, there is no systematic review of the dose-response relationship between acupuncture sessions and its efficacy on PD, but some RCTs have confirmed that the increase of acupuncture sessions may be related to the degree of remission in motor symptoms (Lei et al., 2016). Therefore, it is of certain value to further explore the optimal dose of acupuncture therapy.

In this research, due to the small number of relevant studies, the conclusions drawn are only preliminary analysis, but the effect of acupuncture dose is an important issue that deserves further study. The results of this qualitative and quantitative dose-response meta-analysis found that the combination of acupuncture could significantly improve UPDRS-III scores in PD patients compared with the simple application of medicine and the number of acupuncture sessions was correlated with the reduction of UPDRS-III score. On the one hand, acupuncture session needs to reach a certain dose to obtain better efficacy. In terms of the weekly acupuncture frequency, acupuncture 4 times a week could obtain more obvious improvement in score than acupuncture 2 times a week; the total number of acupuncture session also needs to reach a certain dose to bring about significant improvement in efficacy. On the other hand, acupuncture stimulation may cause the body tolerance. When the number of acupuncture was more than 3 times a week, the improvement of UPDRS-III score significantly increased from 16 to 60 acupuncture sessions, but the score stopped to decrease if the dose continued to increase; the single clinical study of Xu also found that after more than 15 acupuncture session, the improvement on UPDRS-III score was less than before.

This research also found that acupuncture therapy has many characteristics, few side effects, relatively safe, and in the treatment of PD, it has a certain antagonistic effect on the adverse reactions caused by madopar or other dopaminergic drugs, such as nausea and vomiting, which are typical symptoms of the gastrointestinal adverse reactions. Previous study also found that compared with using conventional medication alone, the combination of acupuncture in the treatment of PD, to some extent, can improve clinical safety (Liu et al., 2017). Therefore, it is recommended to use acupuncture in combination with modern medicine when treating PD patients.

### 4.2. Acupuncture session may need to reach a certain dose to obtain better efficacy

Based on the quantitative meta-analysis and the comparative analysis of the two included quantitative RCTs, it can be speculated that both the total acupuncture dose and the weekly frequency of acupuncture may need to reach a certain value to obtain better efficacy. A recent quantitative meta-analysis found no significant improvement in efficacy when the total number of acupuncture treatments was less than 18 times (Xu et al., 2022). According to Figure 4 the clinical effectiveness decreased until 30 acupuncture sessions were reached, which indicated that acupuncture treatment needs to reach a certain dose to obtain better therapeutic effect. So in clinical practice, acupuncture dose should be >30 times to achieve better clinical therapeutic effect. The conclusion in Figure 5 was exactly in line with this, but due to the large dispersion and small number of related studies with acupuncture frequency less than 3 times a week and the quantitative RCT of Li related to low-frequency acupuncture did not find such a large reversal of efficacy, more high-quality quantitative RCTs are needed to verify this result. In addition, comparing the results of Li and Xu, the UPDRS-III score varied significantly before and after high-frequency acupuncture treatment, and no significant difference before and after low-frequency acupuncture treatment, which meant that the improvement of motor symptoms in PD patients was more obvious at the higher weekly frequency of acupuncture. As there were some differences in the baseline between the two studies, more quantitative studies with better homogeneity will be needed to confirm this conclusion. Animal experiments have proved that there is a certain scientific basis for acupuncture to improve motor function in PD, such as reducing neuronal apoptosis of the striatum (Lu et al., 2012), normalizing the brain functional connectivity (Oh et al., 2022), inhibiting the level of lipid peroxides in dopaminergic neurons and protect neurons from oxidative damage (Zuo et al., 2022). However, to the best of our knowledge, there are no multi-session animal experiments to explore the dose-response mechanism of acupuncture in the treatment of PD, so high-quality scientific evidence is still needed.

### 4.3. Acupuncture stimulation may cause the body tolerance

Various acupuncture treatment sessions (twice, three times, four times and so on per week) have been used in acupuncture clinical trials for a total length (periods) of 4, 5, and 8 weeks (Kong et al., 2018; Xu et al., 2020; Hong et al., 2022). This study found that excessive acupuncture stimulation may cause the body to develop a certain tolerance. Acupuncture therapy achieves the purpose of treating diseases by stimulating acupoints on the body surface and mobilizing the inherent regulating function of the body. That means, acupuncture stimulation on acupoints is the start of acupuncture effect, and the generation of acupuncture tolerance may be closely related to it (Xi, 2009). Previous study suggested that the tolerance to endogenous 5-HT may serve as one of the possible mechanisms underlying the development of electro-acupuncture tolerance (Li et al., 1982).

Firstly, in Figure 4, due to the lack of related studies with acupuncture dose more than 80 times and the influence of more small acupuncture dose sample, we cannot speculate that the efficacy will still increase after 80 times. After subgroup analysis, the influence of low acupuncture dose on fitting effect was reduced and Figure 6 (*P* = 0.020) showed a higher goodness of fit than Figure 4 (*P* = 0.067). As shown in Figure 6, when the frequency of acupuncture was at least once every 2 days and the total number of acupuncture exceeded 60 times, the score stopped to decrease, which meant that the clinical efficacy may not be improved as before. It can also be seen that in Figure 6, when acupuncture dose approached 48 times, the efficacy was not as good as before, but it may be the effect of a discrete sample with 48 acupuncture sessions. Secondly, the quantitative clinical study of Xu also found that when acupuncture was applied for 4 times a week, the improvement effect after 15 times of acupuncture treatment was not as good as before. Thirdly, previous quantitative meta-analysis also found that the improvement rate gradually decreased after >36 acupuncture sessions (Xu et al., 2022). Therefore, in clinical practice, considering the economic burden of PD patients, the optimal dose of acupuncture is from 48 to 60 times when the frequency of acupuncture treatment is more than 3 times a week. After the patient have accepted acupuncture treatment for more than 48 times, they can stop acupuncture for a period of time to avoid the body tolerance. Studying the acupuncture frequency with the best clinical efficacy can enable patients to obtain the best clinical efficacy without substantial loss of economic benefit, rather than blindly pursuing high-frequency and long-term treatment. However, there still needs more clinical studies with large samples to explore the impact of high-dose acupuncture treatment on clinical effectiveness.

### 4.4. Feasibility and necessity of this study

Many systematic reviews found that acupuncture therapy showed a moderate or large effect in PD with motor symptoms (Zhou et al., 2020; Kwon et al., 2021). A dose-response meta-analysis on major depressive disorder has found that acupuncture session was strongly correlated with its efficacy (Xu et al., 2022). The published RCT suggests that more acupuncture sessions lead to greater clinical efficacy in motor function of PD (Li et al., 2022). However, to the best of our knowledge, there is currently no published systematic review comparing the effect of the number of acupuncture sessions on the efficacy of PD with motor functions. All indicate that this study has great feasibility and necessity.

### 4.5. Limitations

It should be pointed out that our study also has limitations. Firstly, the definition of dose should include the physical procedures applied in each session, using one or more needles, taking account of the patient's resulting perception (sensory, affective and cognitive) and other responses (including motor) (White et al., 2008). Among them, only the number of acupuncture sessions was compared. Secondly, this study could not account for the individual efficacy of the selected acupuncture points and the results of this study are based on the assumption that different acupuncture points have the same efficacy. Thirdly, more studies with high acupuncture doses are needed to further demonstrate the impact of acupuncture on clinical efficacy. Finally, due to methodological limitations, this study did not examine sources of heterogeneity and publication bias, which may decrease the robustness of the findings.

## Data availability statement

The raw data supporting the conclusions of this article will be made available by the authors, without undue reservation.

## Author contributions

Full access to all of the data in the study and takes responsibility for the integrity of the data and the accuracy of the data analysis: LZ. Concept and design: SL, XL, and JF. Drafting of the manuscript: SL and XX. Critical revision of the manuscript for important intellectual content: SL, XL, JF, and LZ. Statistical analysis: SL. Obtained funding: LZ and JF. Acquisition, analysis, or interpretation of data: All authors. All authors contributed to the article and approved the submitted version.
